# Elucidating the Influence of MPT-driven necrosis-linked LncRNAs on immunotherapy outcomes, sensitivity to chemotherapy, and mechanisms of cell death in clear cell renal carcinoma

**DOI:** 10.3389/fonc.2023.1276715

**Published:** 2023-12-15

**Authors:** Jinbang Huang, Mengtao Liu, Haiqing Chen, Jinhao Zhang, Xixi Xie, Lai Jiang, Shengke Zhang, Chenglu Jiang, Jieying Zhang, Qinhong Zhang, Guanhu Yang, Hao Chi, Gang Tian

**Affiliations:** ^1^ School of Clinical Medicine, Affiliated Hospital of Southwest Medical University, Luzhou, China; ^2^ Pediatric Surgery, Guiyang Matemal and Child Health Care Hospital, Guiyang Children’s Hospital, Guiyang, China; ^3^ School of Stomatology, Southwest Medical University, Luzhou, China; ^4^ First Teaching Hospital of Tianjin University of Traditional Chinese Medicine, Tianjin, China; ^5^ National Clinical Research Center for Chinese Medicine Acupuncture and Moxibustion, Tianjin, China; ^6^ Heilongjiang University of Chinese Medicine, Harbin, China; ^7^ Department of Specialty Medicine, Ohio University, Athens, GA, United States; ^8^ Department of Laboratory Medicine, The Affiliated Hospital of Southwest Medical University, Luzhou, China; ^9^ Sichuan Province Engineering Technology Research Center of Molecular Diagnosis of Clinical Diseases, Luzhou, China; ^10^ Molecular Diagnosis of Clinical Diseases Key Laboratory of Luzhou, Luzhou, China

**Keywords:** MPT-drive necrosis, clear cell renal carcinoma, lncRNAs, tumor microenvironment, drug sensitivity, tumor prognostic model, programmed cell death

## Abstract

**Background:**

Clear cell renal carcinoma (ccRCC) stands as the prevailing subtype among kidney cancers, making it one of the most prevalent malignancies characterized by significant mortality rates. Notably,mitochondrial permeability transition drives necrosis (MPT-Driven Necrosis) emerges as a form of cell death triggered by alterations in the intracellular microenvironment. MPT-Driven Necrosis, recognized as a distinctive type of programmed cell death. Despite the association of MPT-Driven Necrosis programmed-cell-death-related lncRNAs (MPTDNLs) with ccRCC, their precise functions within the tumor microenvironment and prognostic implications remain poorly understood. Therefore, this study aimed to develop a novel prognostic model that enhances prognostic predictions for ccRCC.

**Methods:**

Employing both univariate Cox proportional hazards and Lasso regression methodologies, this investigation distinguished genes with differential expression that are intimately linked to prognosis.Furthermore, a comprehensive prognostic risk assessment model was established using multiple Cox proportional hazards regression. Additionally, a thorough evaluation was conducted to explore the associations between the characteristics of MPTDNLs and clinicopathological features, tumor microenvironment, and chemotherapy sensitivity, thereby providing insights into their interconnectedness.The model constructed based on the signatures of MPTDNLs was verified to exhibit excellent prediction performance by Cell Culture and Transient Transfection, Transwell and other experiments.

**Results:**

By analyzing relevant studies, we identified risk scores derived from MPTDNLs as an independent prognostic determinant for ccRCC, and subsequently we developed a Nomogram prediction model that combines clinical features and associated risk assessment. Finally, the application of experimental techniques such as qRT-PCR helped to compare the expression of MPTDNLs in healthy tissues and tumor samples, as well as their role in the proliferation and migration of renal clear cell carcinoma cells. It was found that there was a significant correlation between CDK6-AS1 and ccRCC results, and CDK6-AS1 plays a key role in the proliferation and migration of ccRCC cells. Impressive predictive results were generated using marker constructs based on these MPTDNLs.

**Conclusions:**

In this research, we formulated a new prognostic framework for ccRCC, integrating mitochondrial permeability transition-induced necrosis. This model holds significant potential for enhancing prognostic predictions in ccRCC patients and establishing a foundation for optimizing therapeutic strategies.

## 1 Introduction

Kidney clear cell carcinoma (ccRCC) represents approximately 3% of all human malignancies and is the predominant tumor affecting the adult kidney, encompassing the majority of renal tumor cases (1). Recent studies have revealed the concerning fact that the incidence of Clear cell renal carcinoma (ccRCC) increases progressively with age, is associated with a number of lifestyle and genetic factors, and is now the 16th most important contributor to cancer-related mortality worldwide (2, 3). Regrettably, the overall survival (OS) and recurrence-free survival rates associated with ccRCC remain suboptimal, necessitating the identification of novel therapeutic targets and prognostic biomarkers. Hence, there is a pressing need to explore and ascertain new avenues for the treatment of ccRCC (4).

Long non-coding RNAs (lncRNAs) are a distinctive class of RNA molecules exceeding 200 nucleotides in length (5, 6). Unlike messenger RNAs (mRNAs), which serve as templates for protein synthesis (7–9). Mounting evidence supports the involvement of dysregulated long non-coding RNAs (lncRNAs) in a wide range of diseases (10, 11). Notably, these lncRNAs display distinct expression patterns that are specific to spatial, temporal, and cell-state contexts, thereby assuming crucial roles in tumorigenesis and the progression of cancer (12). Recently, a growing body of research has identified an expanding repertoire of long non-coding RNAs (lncRNAs) as pivotal regulators in various biological processes. The dysregulation of lncRNAs has been implicated in a wide range of human diseases, as highlighted in several notable studies (13, 14). Emerging studies have highlighted the notable contributions of long non-coding RNAs (lncRNAs) in the pathogenesis of Clear cell renal carcinoma. Among the newly identified programmed cell death pathways, MPT-driven necrosis has emerged as a distinctive mechanism, triggering programmed cell death via specific signaling cascades (15). Therefore, investigating MPT-driven necrosis lncRNAs (MPTDNLs) associated with MPT-driven necrosis holds promising prospects for unveiling novel prognostic approaches and elucidating the immune microenvironment in tumor patients. Furthermore, the utilization of advanced techniques like machine learning can facilitate the development of precise prediction models, thereby bolstering clinical treatment strategies and enabling personalized medicine. Finally, by using qRT-PCR to investigate the function of 15 MPTDNLs, our model’s proficiency in prognostication has been effectively illustrated. It should be underscored, though, that the comprehension of MPT-driven necrosis-associated lncRNAs in ccRCC remains in its formative phase.

As research progresses further, new targets and techniques for immunotherapy continue to emerge. The discovery of MPT-driven necrosis has led to new insights into the formation and progression of tumor diseases (16). MPT-driven necrosis is a regulated cell death triggered by changes in the intracellular microenvironment, including severe oxidative stress and cytoplasmic calcium overload dependent on cyclophilin D (CYPD) (15). Unfortunately, there are few studies on MPT-driven necrosis and Clear cell renal carcinoma. Therefore, we are investigating this aspect. Forecasting models occupy a pivotal position in managing clear cell renal carcinoma, facilitating both clinicians and patients to apprehend probable disease trajectories, thereby tailoring suitable therapeutic approaches. Conventional prognostic models rely heavily on clinical variables, such as age and tumor stage, but typically neglect to adequately integrate molecular biosignatures to cope with the intrinsic molecular heterogeneity of tumors (17, 18). As a result, these models often fail to provide accurate personalized prognostic predictions. To address this issue more comprehensively, modern research is increasingly favoring the integration of molecular biology information into prognostic models to improve their predictive properties and utility. This includes consideration of the molecular subtype of the tumor, mutational load, and gene expression patterns. This integration can provide clinicians and patients with more accurate prognostic information, which can help develop more personalized treatment strategies and improve treatment outcomes (17). Given these limitations, this study aims to develop a new prognostic model that will include the key molecular biology feature of mitochondrial permeability transition-driven necrosis to improve prognostic prediction in Clear cell renal carcinoma. Our goal is to provide more accurate and personalized prognostic predictions with this new model, which will help optimize the management and treatment of Clear cell renal carcinoma.

## 2 Materials and methods

### 2.1 Data sources

The transcriptomic data and corresponding clinical information of ccRCC patients were acquired from the TCGA database (https://portal.gdc.cancer.gov/). Drawing from pertinent literature, the gene set associated with mitochondrial permeability transition-driven necrosis (MPTDN) was identified (19). Patients were then randomly stratified into a training set and a test set at a proportion of 7:3, specifically focusing on genes implicated in MPTDN for ensuing analyses. Utilizing Strawberry Perl software, mRNA was distinguished from lncRNA, with the latter being the subject of interest for subsequent investigations within this study. The clinical variables included in this study encompassed data on patient age, gender, tumor grade, and stage, along with TNM classification (**Supplementary Table 1**).

### 2.2 Screening of MPTDNLs

Through the application of the R package “limma” (20), an analysis was implemented to discern differentially expressed genes among ccRCC patients as represented in the TCGA compendium. The initial phase involved a targeted lncRNA screening, subsequently succeeded by the computation of their correlation with MPT-Drive Necrosis data. The enforcement of a correlative coefficient benchmark of 0.4 (corFilter) and a p-value demarcation of 0.001 (pvalueFilter) facilitated the identification of lncRNAs demonstrating a significant association with the MPTDN data.

### 2.3 Development and validation of prognostic models for MPTDNLs

To amalgamate the survival data of patients with MPTDNLs and renal clear cell carcinoma (ccRCC), we employed the R package “limma” (20). Subsequently, a univariate Cox regression (uni-Cox) (21–23) examination was implemented to discern differentially articulated MPTDNLs that demonstrated a meaningful prognostic correlation (coxPfilter<0.05). Drawing from this outcome, sample randomization into training and test cohorts was undertaken at a 7:3 ratio, facilitated by the R package “caret” (24). Subsequently, a Least Absolute Shrinkage and Selection Operator (LASSO) Cox regression analysis was performed utilizing the R package “glmnet” (25), thereby generating prognostic traits through multivariate Cox (muti-Cox) regression analysis) (21–23). Leveraging these traits, we derived a risk score equation expressed as risk level = Expressed lncRNA1 × CoeflncRNA1 + Expressed lncRNA2 × CoeflencRNA2 +… + Expressed lncRNAn × CoeflincRNAn, with Coefi denoting the correlation coefficient. Within this study, ccRCC patients were stratified into low-risk and high-risk clusters. To evaluate the prognostic predictive capacity of the feature, ROC curves were plotted for various metrics. Internal validation was achieved by displaying ROC curves for both the training and test sets. Comparisons were made between the overall survival rates of the two patient cohorts using Kaplan-Meier survival curves. Moreover, further validation of the influence of clinical variables on patient prognosis was pursued through clinical ROC curves, C-index, and subgroup analyses.

### 2.4 Nomogram construction

To evaluate the proficiency of risk scores as standalone prognostic indicators and subsequently construct related nomograms, analyses invoking both univariate and multivariate Cox regression methodologies were undertaken. In the realm of the TCGA-ccRCC collective, we harnessed the “rms” package (26) within the R software to construct a columnar illustration, embodying risk evaluations concurrent with clinicopathological characteristics, designed to project survival prospects at intervals of 1, 3, and 5 years.

### 2.5 Functional enrichment analysis

Functional enrichment analysis of divergently expressed genes was conducted within the ccRCC scope, intended for annotation and pathway enrichment investigations. Utilizing the clusterProfiler tool (27), an evaluation of Gene Ontology (GO) and KEGG pathways was performed, offering insights into the functionality and pathway involvements of the concerned genes. These insights constitute a foundational guide for subsequent research endeavors.

### 2.6 Immunological analysis of risk characteristics

We employed a suite of algorithms, namely XCELL (28), TIMER (29), QUANTISEQ (30)), MCPCOUNT (31), EPIC (32), CIBERSORT (30), and CIBERSORT-ABS (33) to conduct immuno-infiltration analysis of risk profiles. Moreover, we drew comparisons between the high- and low-risk groups regarding alterations in immune checkpoints.

### 2.7 Targeted drug sensitivity and immunotherapy response prediction

To anticipate the response of ccRCC patients to immunotherapy and their sensitivity to prevalent chemotherapeutic agents, we employed a variety of approaches. Specifically, the R package “oncoPredict” (34) was utilized to conjecture variations in the sensitivity of ccRCC patients to frequently used chemotherapeutic drugs. This package’s analysis was grounded in the half-maximal inhibitory concentration (IC50) data of ccRCC patients sourced from the Genomics of Drug Sensitivity in Cancer (GDSC) database (35).

### 2.8 Cell culture and transient transfection

The human renal clear cell carcinoma cell lines 769-P and HKZ, complemented by the human proximal tubular epithelial cell line HK-2, were propagated in Dulbecco’s Modified Eagle’s Medium (DMEM, GIBCO) supplemented with 10% fetal bovine serum (FBS; Hyclone), 100 U/L penicillin, and 100 mg/L streptomycin (Thermo Fisher). The cellular cultures were maintained at 37°C within an environment of 5% CO2. For transient transfection, Lipofectamine 3000 (Invitrogen, Carlsbad, CA, USA) was used according to the manufacturer’s instructions to transfect Negative Control (NC) and CDK6-AS1 siRNA (RiboBio, Guangzhou, China) into the CRC cells.

### 2.9 qPCR

Total RNA was extracted using the RNA Eazy Fast Tissue/Cell Kit following the manufacturer’s protocols. The cDNA synthesis was achieved using the FastKing RT Kit, following the manufacturer’s suggested procedure. Real-time PCR was performed with the SuperReal PreMix Plus (Sichuan Jielaimei Technology Co., Ltd) reagent, employing the StepOnePlus Real-Time PCR System. The PCR protocol began with a denaturation phase at 95°C for 15 minutes, followed by 40 cycles of denaturation at 95°C for 10 seconds, annealing at 72°C for 20 seconds, and a concluding extension at 60°C for 20 seconds. The primer sequences are detailed in **Supplementary Table 2**.

### 2.10 CCK-8 assay

Cell viability was determined using the Cell Counting Kit-8 (CCK-8) assay. Cells were seeded at a density of 1500 cells per well in 200 µl of complete medium in 96-well plates and cultured at 37°C. After each experiment, 20 µl of CCK-8 reagent (Beyotime, Shanghai, China) was added to each well. The cells were then incubated for an additional 2 hours, and the optical density value (OD450nm) was measured using a microplate reader.

### 2.11 Transwell assay

1×10^5 cells were seeded into either Matrigel-coated (BD Biosciences, San Jose, CA) chambers for invasion assay or uncoated chambers for migration assay. The upper chamber was filled with serum-free medium, while the lower chamber contained complete DMEM medium. After 24 hours of incubation, the cells that migrated or invaded through the membrane were fixed with 4% paraformaldehyde and stained with 0.1% crystal violet. The quantification of cells was performed using a light microscope (Thermo Fisher, Waltham, MA, USA).

### 2.12 Wound healing experiment

To evaluate the migratory capacity of renal clear cell carcinoma cells, a wound healing assay was employed. Transfected cells, housed in six-well plates, underwent incubation at 37°C until reaching approximately 80% confluence. Subsequently, uniform wounds were meticulously generated within the cell monolayer utilizing a 200 μL sterile pipette tip. Following wound induction, cells underwent two rounds of phosphate-buffered saline (PBS) rinsing to eliminate residual material. Subsequently, the culture medium was supplemented with serum-free medium. The progression of cell migration into the wound area was meticulously documented using an Olympus inverted microscope at both the 0 and 24-hour time points.

### 2.13 Plate cloning experiment

In the context of a plate cloning experiment, initial procedures involve the procurement of target cells from a culture vessel to ascertain their physiological robustness. Subsequently, these cells undergo dispersion into singular entities via a mild centrifugation process, ensuring the inception of each clone from an individual cell. The execution of the cloning process necessitates the prior coating of a petri dish or culture plate with a growth medium comprising the requisite constituents, thereby establishing a conducive milieu for cellular proliferation. Following the coating, dispersed cells are introduced onto the prepared medium at a density deemed suitable for the intended purpose. Thereafter, the petri dishes or plates are positioned within a cell culture incubator and subjected to cultivation under conditions encompassing optimal temperature and controlled CO2 levels.

### 2.14 Statistical analysis

Statistical assessments were accomplished with R software version 4.2.3 and Strawberry Perl version 5.30.0. For juxtaposing overall survival (OS) between cohorts of high-risk and low-risk, both Kaplan-Meier (KM) survival trajectories and the log-rank evaluation were utilized. The predictive potential of correlation features, as constructed by the LASSO Cox regression model, was evaluated via ROC curves. We examined the differential representation of tumor-infiltrating immune cells, immune checkpoints, and immune function between the groups via the Wilcoxon test. Differences were deemed statistically significant when p < 0.05 and FDR < 0.05. Results from the CCK-8 assay were analyzed with GraphPad Prism Software (version 8.3.0). Data, representing the means ± standard deviation (SD) from three independent investigations, were scrutinized through analysis of variance (ANOVA). A p-value less than 0.05 was designated as indicative of statistical significance.

## 3 Results

### 3.1 Identification of candidate mitochondrial permeability transitions driving necrosis-associated LncRNAs

**Figure 1:**
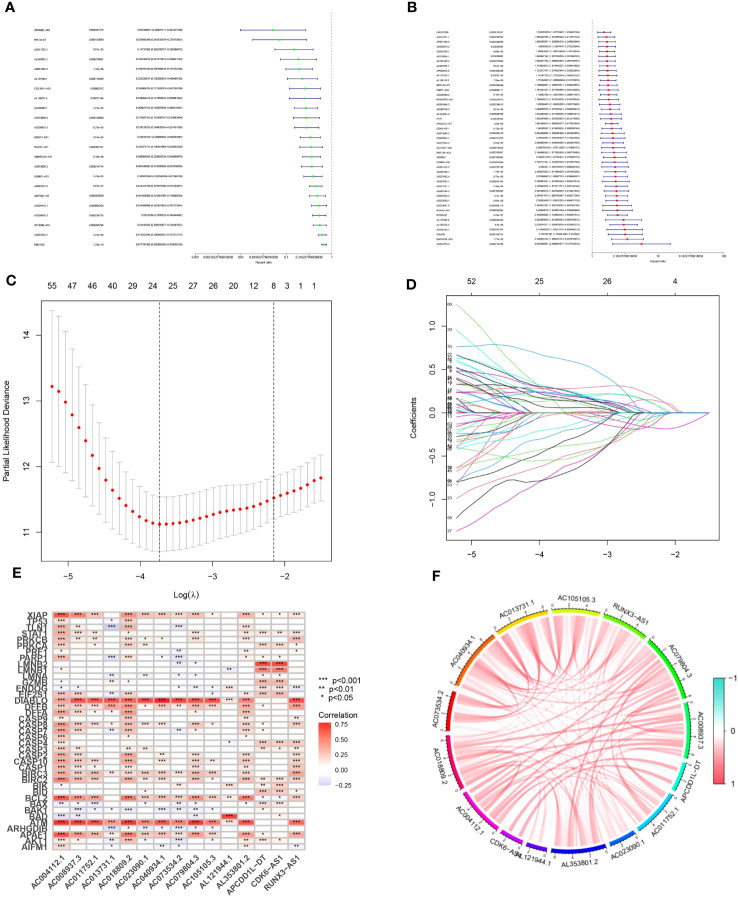
Isolation of Candidate Long Non-coding RNAs (lncRNAs) Associated with Mitochondrial Permeability Transition-Driven Necrosis. **(A, B)** Univariate Cox regression analysis executed to evaluate the prognostic significance of lncRNAs implicated in mitochondrial permeability transition-driven necrosis. **(C)** Tenfold cross-validation facilitates the fine-tuning of parameter selection in the Lasso model. **(D)** Visualization of Lasso coefficient trajectories. **(E)** Heatmap illustrating the correlation matrix between the 15 lncRNAs and the genes implicated in mitochondrial permeability transition-driven necrosis. **(F)** Exploratory correlation analysis conducted on 15 lncRNAs linked with mitochondrial permeability transition-driven necrosis. Statistical significance is denoted as *p<0.05; **p<0.01; ***p<0.001.

Our findings revealed the identification of 1437 long non-coding RNAs (MPTDNLs) linked with mitochondrial permeability transition-driven necrosis. These identified lncRNAs underwent a univariate Cox analysis, allowing the differentiation of low-risk and high-risk lncRNAs associated with this condition (**Figures 1A, B**). Implementing the Lasso algorithm enabled a deeper analysis of these lncRNAs, whereby we pinpointed the juncture with the smallest cross-validation error via Lasso regression cross-validation. This procedure culminated in the segregation of 24 lncRNAs, with their respective regression coefficients and cross-validation patterns subsequently subjected to analysis (**Figures 1C, D**). We then employed a multifactor Cox proportional hazard regression model to streamline this high-dimensional data, resulting in the final identification of 15 pertinent MPTDNLs; AC004112.1, AC008937.3, AC011752.1, AC013731.1, AC018809.2, AC023090.1, AC040934.1, AC073534.2, AC079804.3, AC105105.3, AL121944.1, AL353801.2, APCDD1L-DT, CDK6-AS1, and RUNX3 -AS1, along with their respective regression coefficients, including 0.4138, 0.4786, 0.4745, and 0.4086. The linear prediction model was constructed from these 15 MPTDNLs’ weighted regression coefficients within the multivariate Cox analysis. Herein, the risk score was calculated as risk score = (regression coefficient of MPTDNLs1 × expression level of MPTDNLs1) + (regression coefficient of MPTDNLs2 × expression level of MPTDNLs2) +…… + (regression coefficient of MPTDNLsn × expression level of MPTDNLsn). Further investigation revealed a robust correlation between genes associated with mitochondrial permeability transition-driven necrosis and the MPTDNLs (**Figure 1E**). Moreover, the 15 MPTDNLs exhibited strong intercorrelations (**Figure 1F**).

### 3.2 Model construction and validation for disease prediction

**Figure 2:**
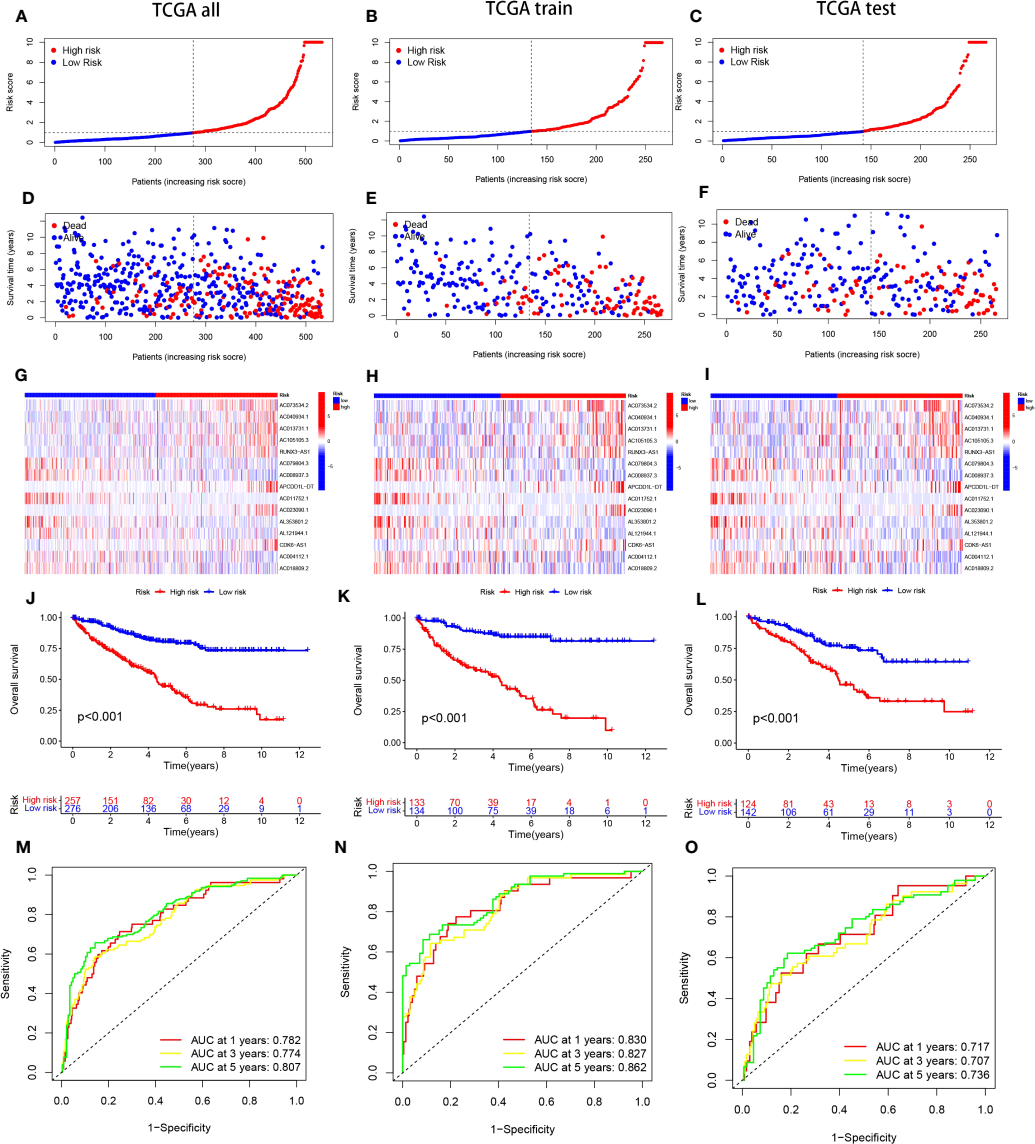
portrays the Kaplan-Meier (KM) survival estimates for the overall data, as well as for the distinct training and testing cohorts from the constructed risk model. Subfigures **(A-C)** delineate the distribution of risk scores assigned to ccRCC patients. Concurrently, subfigures **(D-F)** encapsulate the dispersions of survival duration and status across the low- and high-risk categories of ccRCC patients. The differential expression of the selected 15 lncRNAs is illustrated via heatmaps within subfigures **(G-I)**. Moreover, the overall survival (OS) trajectories for patients classified as high or low risk across the distinct groups are depicted in subfigures **(J-L)**.In a bid to gauge the sensitivity and precision of the prognostic model, the area beneath the curve for survival estimates at one, three, and five years across the varied groups was calculated, as highlighted in subfigures **(M-O)**.

The predictive model was developed by segregating the samples into training and verification cohorts in a 7:3 ratio. The training cohort underpinned the model’s formulation, whereas the verification set served to evaluate the model’s precision. Risk scores for individual specimens were derived by aggregating the product of the expression levels of the 15 chosen necrosis-related lncRNAs and their corresponding regression coefficients. Subsequent to the classification of samples into high- and low-risk groups, predicated on the dataset’s median value, correlative training and validation cohorts were established. This methodology facilitated the computation of risk scores for every specimen within the TCGA collective. The risk scores of ccRCC patients across the trio of datasets were arranged in order, yielding scatter plots that mirrored survival status. An observed trend demonstrated increased mortality rates with elevated risk scores in the TCGA cohort of ccRCC patients (**Figures 2A-F**). The “pheatmap” package in R was deployed to generate heatmaps (**Figures 2G-I**), which displayed the distribution of 15 OS-associated lncRNAs’ expression profiles among high- and low-risk cohorts. In the lower risk group, a significant inverse correlation was observed in eight lncRNAs, while they exhibited a positive correlation in the high-risk group, marking them as high-risk lncRNAs.The residual seven lncRNAs seemed to confer a protective function. To ascertain the prognostic significance of our risk score model for ccRCC patients, we probed the interplay among survival prognosis, survival span, and the stratification of patients into elevated- and diminished-risk groups. Kaplan-Meier analysis was employed to draft survival trajectories, revealing marked variances (P<0.05) between the elevated- and diminished-risk groups throughout the comprehensive dataset (**Figure 2J**), as well as within the validation (**Figure 2K**) and training segments (**Figure 2L**).In the diminished-risk group, a more favorable overall survival was noted compared to the elevated-risk cohort. The model’s performance in ccRCC patients was gauged by plotting ROC curves. The AUC values indicated the predictive accuracy of the model, with larger values indicating superior performance. The findings substantiated that the model possessed noteworthy predictive prowess and sensitivity concerning patient prognosis, presenting AUC values of 0.782, 0.774, and 0.807 for survival at 1-year, 3-year, and 5-year intervals, correspondingly (**Figure 2M**). To further validate the model’s accuracy, internal validation of the randomly grouped training and testing sets was performed via ROC analysis. The AUCs after 1, 3, and 5 years were 0.830, 0.827, 0.862 for the training set, and 0.717, 0.707, 0.736 for the test set, substantiating the model’s efficacy in survival prediction (**Figures 2M-O**).

### 3.3 Principal component analysis of all genes, MPT-driven necrosis-associated genes, MPT-driven necrosis-associated lncRNAs, and model lncRNAs

**Figure 3:**
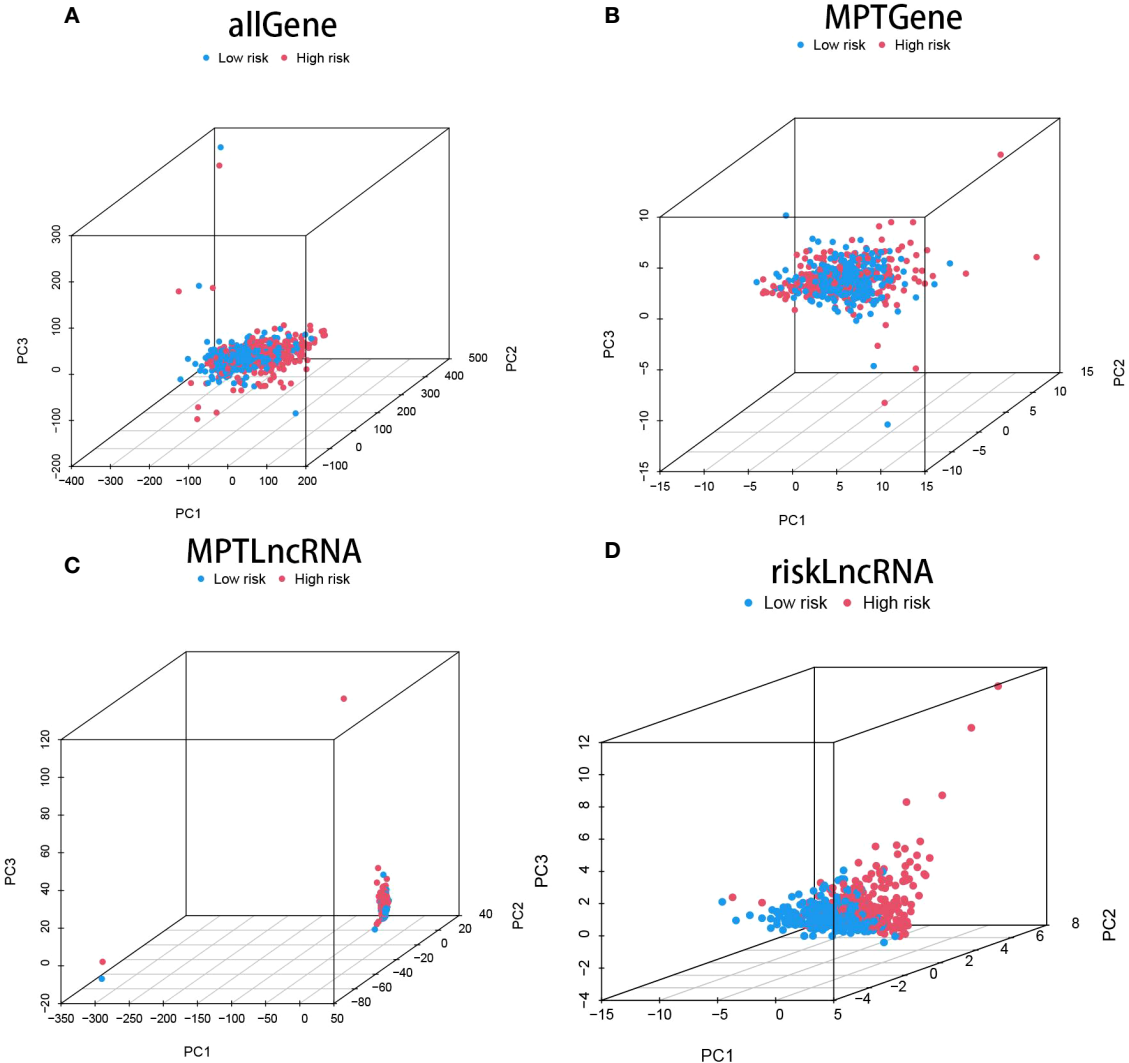
showcases the Principal Component Analysis (PCA) plot for all the genes **(A)**, the PCA plot specifically for genes implicated in mitochondrial permeability transition-driven necrosis (MPT) **(B)**, the PCA plot for lncRNAs associated with MPT-driven necrosis **(C)**, and finally, the PCA plot representing risk-associated lncRNAs **(D)**.

Drawing upon the principal component analysis of our risk model, we discerned disparities across all genes, genes linked to mitochondrial permeability transition-driven necrosis, lncRNAs pertinent to mitochondrial permeability transition-driven necrosis, and lncRNAs associated with risk (**Figures 3A-D**). Remarkably, within the risk lncRNAs, substantial divergences were perceptible between the elevated-risk and diminished-risk assemblies, which were efficiently partitioned into two unique, relatively self-contained clusters (**Figure 3D**). These findings attest to the robustness of our methodological strategy in differentiating between cohorts of diminished and heightened risk, emphasizing the stability, extensive versatility, and predictive prowess of the established risk framework.

### 3.4 Correlative analysis of MPTDNLs with clinicopathological features

**Figure 4:**
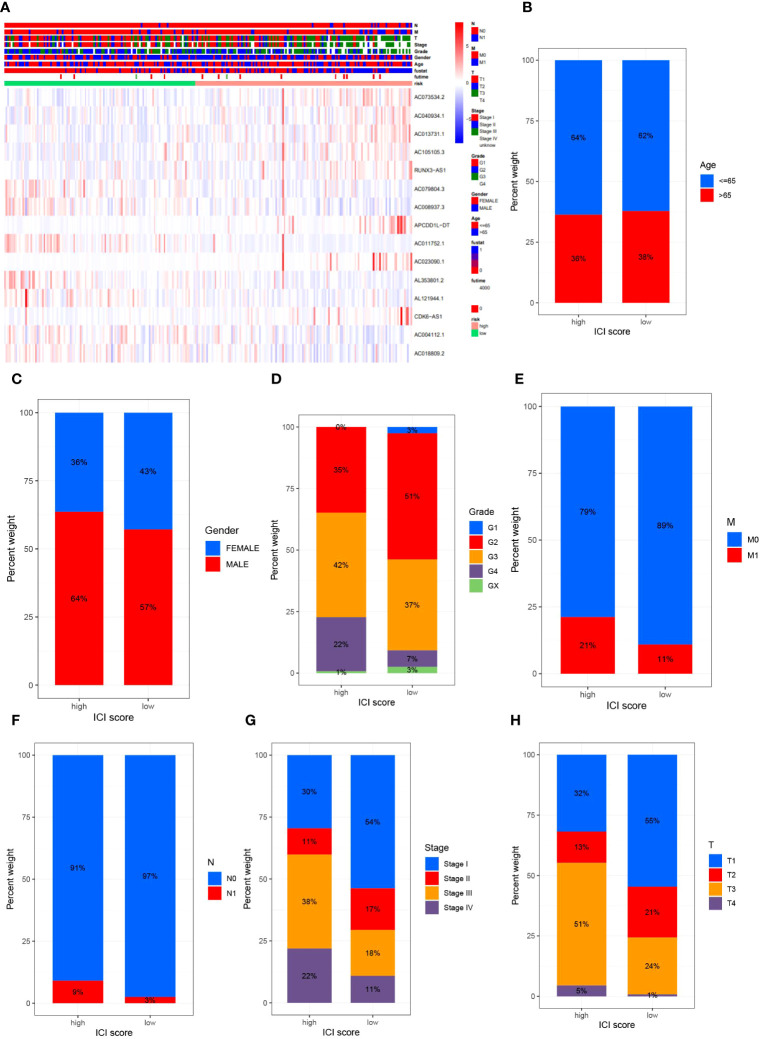
delineates the outcomes of the correlational study between MPTDNLs and clinicopathologic characteristics. Subfigure **(A)** exhibits a heatmap illuminating the association between risk scores and clinicopathologic traits. Figures **(B-G)** explicate the variance in patient counts within high- and low-risk categories as they pertain to diverse pathological attributes, including age **(B)**, gender **(C)**, tumor grade **(D)**, M-stage **(E)**, N-stage **(F)**, overall cancer stage **(G)**, and T-stage **(H)**.

To elucidate the relationship between high- and low-risk categories and various clinical attributes, we generated correlation heatmaps that exhibited the interconnection between risk groups and variables such as age, sex, tumor grade, stage, T-stage, N-stage, M-stage, and risk score (**Figure 4A**), incorporating data from all TCGA renal clear cell carcinoma patients. Additionally, we delved into the variances in high and low-risk group distributions across diverse clinical attributes, including age, gender, tumor grading, staging, as well as T, M, and N designations. Concurrently, we discerned dissimilarities in the patient population bearing different clinicopathologic features within high- and low-risk groups. The 15 MPTDNLs noticeably impacted the prevalence of certain clinicopathologic attributes (**Figures 4B-H**).

### 3.5 Clinical subgroup analysis of MPT-driven necrosis-associated lncRNA models

**Figure 5:**
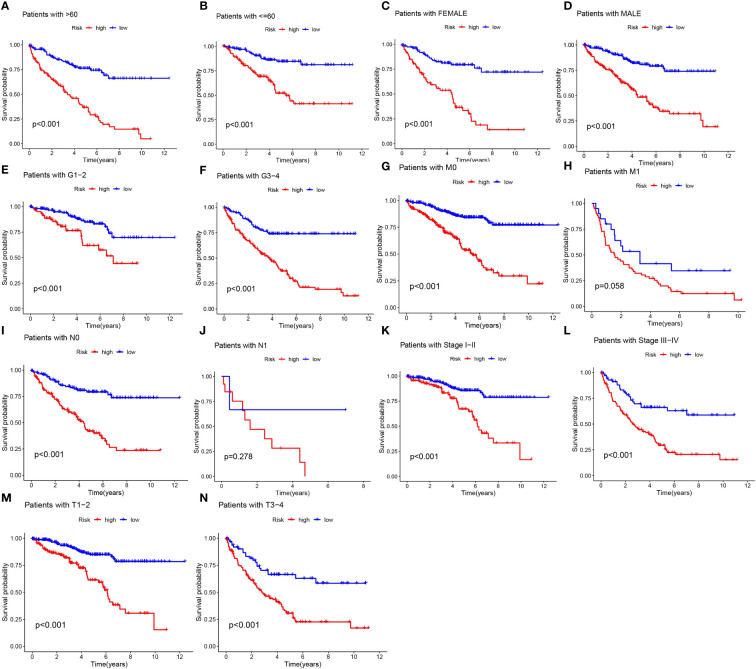
Under different clinical characteristics, we plotted Kaplan-Meier curves **(A-N)** for ccRCC patients in the low and high risk groups.

Acknowledging the substantial variances in individual clinical determinants of OS across high-risk and low-risk cohorts, and intending to delve deeper and juxtapose whether prognostic outcomes shift among distinct clinical subsets, we partitioned ccRCC patients into seven diverse subgroups founded on their clinical attributes. Survival curve discrepancies between high- and low-risk cohorts were scrutinized and compared via analysis and juxtaposition of varying subsets about age (greater than 60 and 60 or below), gender (male and female), tumor grade (G1-G2 and G3-4), M-stage (M0 and M1), N-stage (N0 and N1), clinical stage (I-II and III-IV), and T-stage (T1-T2 and T3-T4) (**Figures 5A–N**). We could discern that the overall survival of high-risk patients markedly trailed that of the low-risk patients in all subgroups barring patients with M1 and N1 stages, implying a survival advantage for the low-risk patients. From the scrutiny of these results, we inferred that our MPTDNLs risk framework acts as a steadfast and reliable prognostic tool in the clinical setting, adept at precisely anticipating the prognosis of distinct clinical ccRCC subcategories.

### 3.6 Independent prognostic analysis of clinical characteristics with nomograms

**Figure 6:**
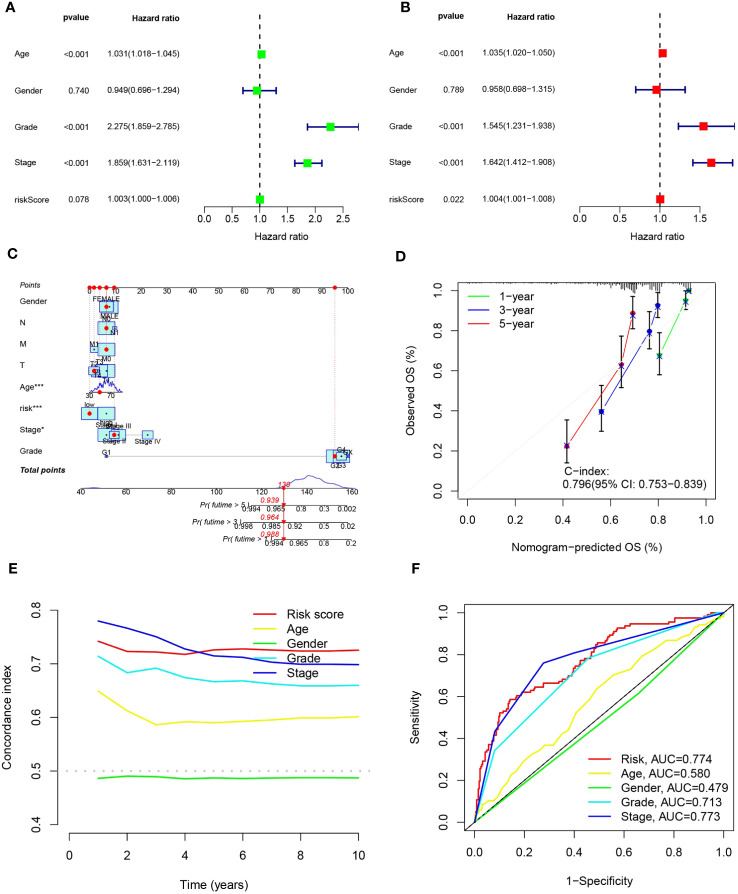
portrays the construction of the column-line schematic and the outcomes of the analysis related to independent prognostic determinants. **(A, B)** showcase the findings of univariate and multivariate COX regression assessments. **(C)** presents column-line representations evaluating one-, three-, and five-year OS for patients afflicted with ccRCC. **(D)** depicts calibration curves tailored to the column diagrams. **(E)** exhibits c-index trajectories for diverse characteristics. Lastly, **(F)** illustrates ROC curves pertinent to varying features. Statistical significance is denoted as *p<0.05, ***p<0.001.

Considering the compelling link between the prognostic risk schema we formulated and negative prognosis, we implemented both univariate and multivariate independent prognostic analyses, integrating risk scores with common clinical traits for each ccRCC patient. The goal was to ascertain whether these 15 MPTDNLs could act as independent prognostic determinants. Univariate analysis revealed a significant relationship with the prognosis of ccRCC patients concerning age, tumor grade, and clinical stage (**Figure 6A**). Multivariate Cox analysis further highlighted associations with age, gender, tumor grading, clinical staging, and the risk score. This evaluation reinforced age, tumor grade, clinical stage, and risk score as reliable and independent prognostic indicators(p-value <0.05)(**Figure 6B**). To enhance the clinical feasibility and usefulness of the established risk schema, we created a column-line diagram, employing gender, N stage, M stage, T stage, age, risk score, staging, and grading as instruments to project the prognostic probabilities of survival at 1-, 3-, and 5-year intervals (**Figure 6C**). The risk score wielded the most significant influence on the prediction of OS, suggesting that the prognosis of ccRCC could be forecasted with greater precision through this risk model. The calibration curves exhibited a noteworthy alignment between projected and actual outcomes regarding the OS probability at 1, 3, and 5 years, indicating the robust stability of the Nomogram plot (**Figure 6D**). The risk score’s c-index value and the area beneath the ROC curve outperformed other clinical metrics, further validating the superior predictive capability of our established model over other parameters in estimating patient survival (**Figures 6E, F**).

### 3.7 Enrichment analysis of ccRCC patients based on prognostic markers

**Figure 7:**
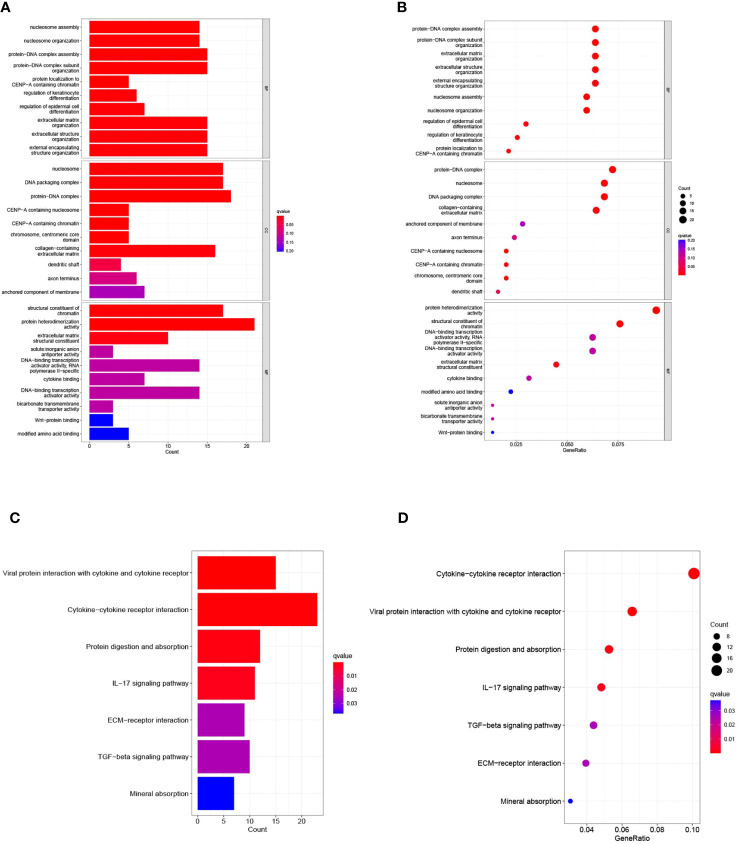
presents the results of the Gene Ontology (GO) and Kyoto Encyclopedia of Genes and Genomes (KEGG) pathway enrichment analyses. **(A)** Bar graph illustrating the ten most prominent terms in GO enrichment. **(B)** Bubble plots displaying the leading ten enriched terms within GO. **(C)** Histogram of the seven most recurrent terms within KEGG enrichment. **(D)** Bubble chart highlighting KEGG’s seven most frequently encountered terms.

To elucidate the relationship between biological processes, signal transduction pathways, and risk scores, we utilized GO functional analysis and KEGG enrichment analysis on differentially expressed genes within elevated- and diminished-risk collectives. Thresholds for markedly amplified items were defined at logFCfilter=1 and fdrFilter=0.05, generating distinct enrichment results in both risk assemblies. GO enrichment analysis revealed that the most influential biological processes (BPs) included protein-DNA complex assembly, organization of protein-DNA complex subunit, extracellular matrix, external encapsulated structure organization, among others. Cellular components (CC) were predominantly protein-DNA complexes, DNA packaging complexes, and nucleosomes, etc. Protein heterodimer activity and structural constituents of chromatin largely represented the molecular function (MF) (**Figures 7A, C**). KEGG enrichment underscored the major roles of cytokine-cytokine receptor interactions, viral proteins in tandem with cytokine-cytokine receptor interactions, protein digestion and absorption, and the IL-17 signaling pathway (**Figures 7B, D**).

### 3.8 Correlative predictive analysis of immune cell infiltration and tumor microenvironment by MPT-driven necrosis-associated LncRNAs models

**Figure 8:**
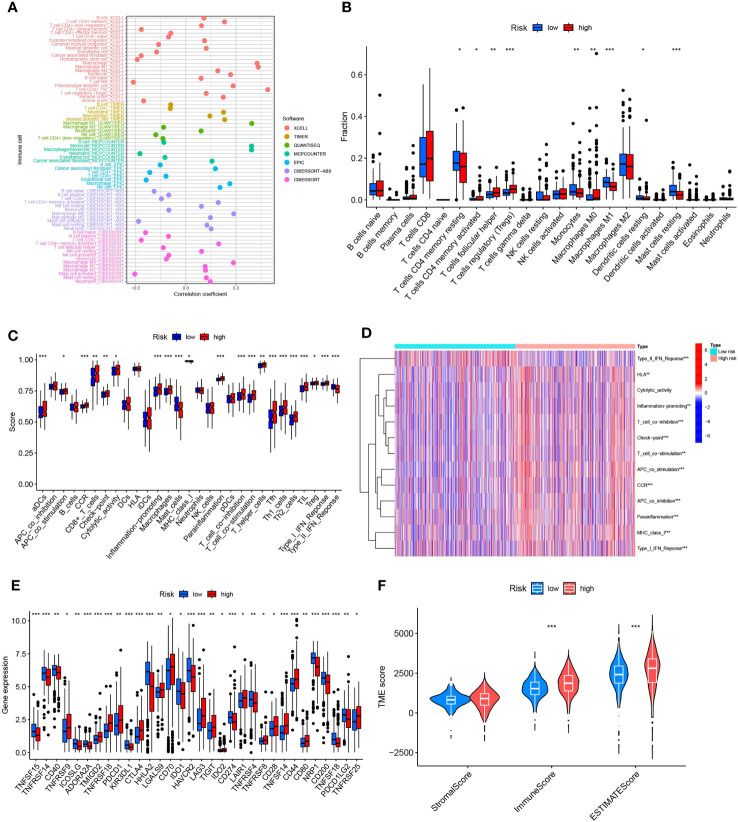
Illustrates the predictive capacity of MPTDNLs risk scores for the tumor microenvironment and immunotherapy. **(A)** Bubble chart representing the abundance of immune cells. **(B)** Depicts discrepancies in immune cell infiltration between high- and low-risk groups. **(C, D)** Contrast of immune function disparities between the high- and low-risk cohorts. **(E)** Highlights variations in immune checkpoint expression between high and low-risk groups. **(F)** Portrays the differences in tumor microenvironment scores between the high- and low-risk groups. Statistical significance is denoted by *p<0.05; **p<0.01; ***p<0.001.

The intricate interaction between neoplastic cells and the tumor microenvironment (TME) is intrinsically linked with cancer expansion, infiltration, and metastasis. Tumor-infiltrating immune cells (TIICs), integral to the TME, exhibit a composition and distribution intrinsically linked to tumor advancement (36). Initially, we probed the linkage between risk scores and TIICs abundance leveraging seven distinct algorithms: XCELL, TIMER, QUANTISEQ, MCPCOUNTER, EPIC, CIBERSORT-ABS, and CIBERSORT. Primarily, a positive relationship was detected between immune cell infiltration and risk scores within the algorithm, particularly with regard to T cells CD4+ Th2, M1 macrophages, and general macrophages (**Figure 8A**). Appreciating the significance of immune cells in immunotherapy, we scrutinized differences in immune cell composition between high- and low-risk cohorts. Several immune cell types exhibited significant discrepancies between the risk groups, including T-cell CD4 memory quiescent, T-cell CD4 memory activated, T-cell follicular helper, T-cell regulatory cells (Tregs), monocytes, macrophage M0, macrophage M1, quiescent dendritic cell, and dormant mast cells. Within this set, activated memory T cell CD4, follicular helper T cells, regulatory T cells (Tregs), and M0 macrophages were more prominent in the high-risk group while quiescent memory T cell CD4, monocytes, M1 macrophages, quiescent dendritic cells, and dormant mast cells were more prevalent in the low-risk group (**Figure 8B**). Recognizing the crucial role of immune functionality, we undertook a single-sample Gene Set Enrichment Analysis (ssGSEA) focused on immune function. Distinct immune function scores, including CD8+ T cells, pro-inflammatory cells, and macrophages, were markedly increased in the diminished-risk group. Conversely, Mast cells, MHC class I, and Type II IFN Response scores prevailed in the elevated-risk group (**Figures 8C, D**). Throughout our research, marked differences in immune checkpoint expression between the risk groups were observed, underlining the pivotal role of immune checkpoints in modulating immune system functionality. Intriguingly, all 32 immune checkpoint genes demonstrated significant variation (p<0.05). In the low-risk cohort, 15 immune checkpoint genes (TNFSF15, TNFRSF14, CD40, ICOSLG, ADORA2A, KIR3DL1, HHLA2, IDO1, HAVCR2, CD274, TNFRSF4, NRP1, CD200, TNFSF18, and PDCD1LG2) were markedly up-regulated, whereas the remaining 17 were substantially down-regulated, indicating a potential increased significance for checkpoint-based immunotherapy in therapeutic strategy (**Figure 8E**). Lastly, a comprehensive analysis of the tumor microenvironment unveiled distinct differences in immune cell scores and immune-stromal cell composite scores between the high- and low-risk groups. We discerned that both the immune cell score and the immune-stromal cell composite score were elevated in the high-risk group, suggesting an increased stromal cell content (**Figure 8F**).

### 3.9 Differential analysis of drug sensitivity of LncRNAs associated with mitochondrial permeability transition-driven necrosis

**Figure 9:**
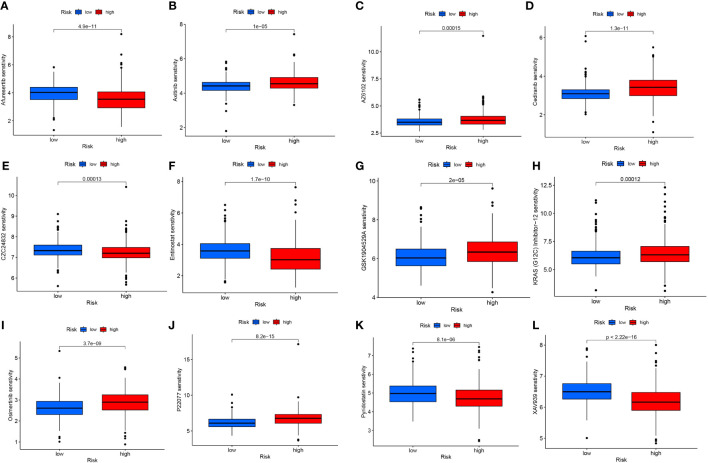
Differences in IC50 of different immunotherapy drugs according to risk score: **(A)** Afuresertib, **(B)** Axitinib, **(C)** AZ6102, **(D)** Cediranib, **(E)** CZC24832, **(F)** Entinostat, **(G)** GSK1904529A, **(H)** KRAS (G12C) Inhibitor-12, **(I)** Osimertinib, **(J)** P22077, **(K)** Pyridostatin, **(L)** XAV939.

The application of risk scores facilitates a comprehensive assessment of immunotherapy effectiveness in ccRCC patients and assists in adjusting medication dosages. Among the 12 immunotherapeutic agents employed for ccRCC treatment, there was a notable difference in drug sensitivity between the elevated-risk and diminished-risk collectives (p<0.05). Five medications - Afuresertib, CZC24832, Entinostat, Pyridostatin, and XAV939 - exhibited lower IC50 values in the elevated-risk group than in the diminished-risk group, suggesting heightened sensitivity in the elevated-risk group (**Figures 9A, E, F, K, L**). Conversely, the remaining seven drugs - Axitinib, AZ6102, Cediranib, GSK1904529A, KRAS (G12C) Inhibitor-12, osimertinib, and P22077 - demonstrated higher IC50 values in the elevated-risk group, indicating a diminished sensitivity in the elevated-risk group compared with the diminished-risk group (**Figures 9B-D, G-J**). The employment of risk scores enables an exhaustive study of immunotherapy response in ccRCC patients, thereby enhancing the accuracy of pharmacological interventions.

### 3.10 CDK6-AS1 plays a key role in stimulating the proliferation and migration of renal clear cell carcinoma cells

**Figure 10:**
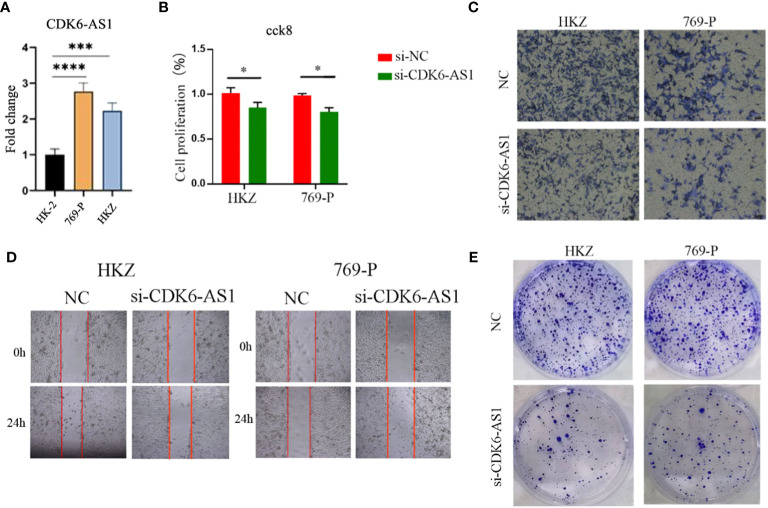
CDK6-AS1 has been demonstrated to boost the proliferation, invasion, and migration of renal clear cell carcinoma cells, as determined through a range of analyses: **(A)** qPCR assessment, **(B)** CCK-8 analysis, **(C)** Transwell examination, **(D)** wound healing assay, and **(E)** plate cloning assay. * denotes p-value <0.05, *** denotes p-value <0.001, **** denotes p-value <0.0001.

The upregulation of CDK6-AS1 in renal clear cell carcinoma cells was substantiated through quantitative polymerase chain reaction (qPCR), aligning consistently with the outcomes of our bioinformatics analysis (**Figure 10A**). To comprehensively investigate the potential involvement of CDK6-AS1 in renal clear cell carcinoma, a battery of *in vitro* experiments was conducted. Notably, the CCK-8 assay demonstrated a significant reduction in cellular proliferative capacity upon CDK6-AS1 silencing (**Figure 10B**). Subsequent findings from transwell assays underscored the consequential inhibition of cell invasion and migration capabilities following interference with CDK6-AS1 expression (**Figure 10C**). The scratch assay results revealed the facilitative role of heightened CDK6-AS1 expression in tumor cell migration (**Figure 10D**), while the plate cloning assay corroborated that diminished CDK6-AS1 expression restrained the proliferation and invasion potential of tumor cells (**Figure 10E**). Collectively, these observations unveil the oncogenic properties attributed to CDK6-AS1, elucidating its pivotal role in driving the proliferation, invasion, and migration of renal clear cell carcinoma.

## 4 Discussion

Kidney clear cell carcinoma (ccRCC) represents a prevalent histological subtype of renal cell carcinoma, bearing a dismal prognosis and posing a significant societal health burden (37–39). Addressing the challenge of predicting disease progression and optimizing treatment strategies has emerged as a critical focus in research (40). Evidence suggests that lncRNAs are associated with malignant traits in ccRCC, including tumor growth, invasion, and metastasis, potentially contributing to tumorigenesis (40, 41). Additionally, in the context of tumors, disturbances in mitochondrial function, specifically mitochondrial permeability transition (MPT), lead to a depletion of mitochondrial DNA and RNA, thereby impeding the activity of the mitochondrial respiratory chain, which ultimately culminates in tumor cell death (42–46). MPT drives a rapid increase in the permeability of the inner mitochondrial membrane. This ultimately leads to dissipation of the mitochondrial membrane potential, uncoupling of the respiratory chain, and entry of water and ions, which triggers osmotic swelling of the mitochondrial matrix, ultimately leading to mechanical rupture of the outer mitochondrial membrane.The pronounced manifestation of the MPT has the potential to bring about cell death by regulating the necrotic or apoptotic pathway (45–47). Studies have proposed that mitochondria-targeted therapies hold promise in restraining cancer cell proliferation by modulating the mitochondrial redox state or promoting cancer cell apoptosis via MPT regulation (48). Although targeted therapies such as sunitinib, sorafenib, bevacizumab, and ticlosimab demonstrate some efficacy against renal clear cell carcinoma, their sensitivity remains limited, necessitating the continued reliance on surgical interventions as the primary treatment modality (49–51). Recently, investigations have suggested that inducing MPT could serve as a novel approach to the development of innovative cancer therapies by promoting mitochondria-mediated cell death and inhibiting cancer cell differentiation (52). Thus, to further comprehend ccRCC pathogenesis, forecast tumor prognosis, and establish a foundation for immunotherapy and drug-based interventions, we seek to construct a model involving mitochondrial permeability transition-driven necrosis-associated lncRNAs.

Initially, we harnessed transcriptomic and clinical data from ccRCC patients accessible through the TCGA database, integrating it with mitochondrial permeability transition-driven necrosis (MPTDN)-associated genomes. Employing Lasso regression analysis and Cox proportional hazards regression analysis, we identified 15 independent MPTDNLs as prognostic variables uniquely pertinent to ccRCC. Subsequently, we stratified the patients into elevated-risk and diminished-risk categories, followed by evaluations on immune-associated metrics and drug responsiveness. Remarkably, we observed substantial distinctions between the two groups, consolidating the clinical relevance of our risk model. Furthermore, we undertook clinicopathologic feature correlation analysis and clinical subgroup analysis, enabling the accurate prognosis prediction for distinct clinical subgroups within the ccRCC cohort. These significant findings furnish a robust theoretical foundation, empowering clinicians with the means to render more precise decisions and enhance the overall survival quality of ccRCC patients.

The elevated expression of APCDD1L-DT in renal clear cell carcinoma cells has piqued the scientific community’s interest. This heightened expression potentially correlates with tumor development and progression, given the pivotal role that aberrant gene expression levels often play in the genesis and dissemination of malignant neoplasms. This influence extends to potential modulation of tumor proliferation, apoptosis, and angiogenesis as pivotal biological processes governing tumorigenesis. On a related note, AC011752.1 presents an avenue of inquiry pertaining to its involvement in the etiology of renal clear cell carcinoma and its impact on the efficacy of immunotherapeutic interventions. Although immunotherapy has emerged as a cornerstone in the therapeutic arsenal against renal clear cell carcinoma, it remains an incontrovertible fact that not all patients manifest a favorable response to this treatment modality. Consequently, unraveling the intricacies of AC011752.1 and similar biomarkers within the context of immunotherapy offers the promise of discerning patient responses and, in turn, refining the tailoring of individualized therapeutic regimens (53, 54). Furthermore, AC008937.3, AC018809.2, AC023090.1, CDK6-AS1, and AC073534.2 were also found to feature prominently in prognostic assessments of lung cancer (55), bladder cancer (56), hepatocellular carcinoma (57), gastric cancer (58), and acute myeloid leukemia (59), further underscoring their relevance in disease progression predictions across diverse malignancies. Remaining MPTDNLs, while yielding limited information from existing research findings, harbor untapped potential for predictive value, warranting future investigation and exploration.

The tumor microenvironment (TME) is a complex network encompassing tumor cells, mesenchymal stromal cells, blood vessels, extracellular matrix, and growth factors, all collaboratively influencing tumor proliferation, invasion, and metastasis (60, 61). Given the TME’s heterogeneity and ongoing cross-talk with tumor cells, its composition stands as a pivotal prognostic determinant in cancer, while simultaneously governing the responsiveness to emerging immunotherapies (62).In this investigation, we discovered significant and positive associations between the risk scores and T cell CD4+ Th2, as well as macrophage M1 and M2 infiltration within the ccRCC immune cells. High PCIF1 expression exhibited a positive correlation with CD4+ T cell infiltration specifically in renal clear cell carcinoma (63). Macrophages found in primary or secondary tumor tissues are commonly designated as tumor-associated macrophages (TAMs), representing the predominant macrophage population within the tumor microenvironment (64). These macrophages typically exhibit a Th1-responsive gene expression profile and are proficient in cytokine secretion while presenting MHC II and B7 molecules, thereby effectively facilitating antigen presentation. This immune machinery serves the dual purpose of defending against pathogenic incursion, surveilling tumor pathogenesis, and inducing Th1 immune responses within the macrophage population (64, 65). Additionally, the macrophage M2 co-expression factor demonstrated a correlation with the immune microenvironment, rendering it a promising prognostic indicator for renal clear cell carcinoma (64). Notably, macrophages emerged as the predominant immune cells within the tumor microenvironment of renal clear cell carcinoma, with changes in their phenotypic profile manifesting as potential indicators of unfavorable clinical outcomes (66).

In this investigation, we conducted a comprehensive analysis of immune checkpoints within the tumor microenvironment, revealing significant distinctions between the two risk groups. These findings underscore the pivotal role of immune checkpoints in modulating immune system function and their potential significance in tumor therapy. Among the 32 immune checkpoint genes scrutinized, all exhibited notable differences in expression levels between the two risk groups. Remarkably, the high-risk group displayed elevated immune cell scores and stromal cell immune cell composite scores, indicative of heightened stromal cell content within the tumor microenvironment. Such observations potentially link increased stromal cell presence to tumor malignancy and prognosis. These immune checkpoint study outcomes hold profound importance for the advancement of immunotherapy, which has emerged as a pivotal approach in tumor therapy. Immune checkpoint inhibitors have garnered substantial attention as a therapeutic strategy, with notable immune checkpoints, including CTLA4, PD-1, PD-L1, and LAG3, identified as effective targets for stimulating T cell-mediated anti-tumor immunity (67). The significance of PD-L1 in oncology has undergone extensive investigation, owing to its status as a pivotal immune checkpoint mechanism (68), which serves to curtail hyperactivation of the immune response via its interaction with the programmed death receptor 1 (PD-1) situated on activated lymphocytes (69, 70). It is noteworthy that chemotherapy has been observed to augment PD-L1 expression through diverse proliferative pathways (69). Furthermore, immune checkpoint inhibitors, encompassing those targeting CTLA-4 and PD-1, have demonstrated their capacity to incite tumorigenic reactions within the realm of renal clear cell carcinoma (71, 72).

The study of chemosensitivity in ccRCC is of great importance, especially in the construction of prognostic models and precision medicine. Our study successfully categorized patients with ccRCC into two groups, high-risk and low-scoring risk, based on the MPTDNLs signature, which provides critical information for more targeted treatment decisions. Regarding the analysis of chemotherapy sensitivity, we further investigated the difference in response between several common chemotherapeutic agents in high- and low-scoring risk patients. This study not only helps to identify which patients are more likely to benefit from chemotherapy but also provides strong support for the implementation of precision medicine. By gaining insight into the chemotherapy sensitivity of ccRCC patients, physicians can better personalize treatment regimens to increase treatment efficacy, reduce unnecessary drug exposure, and mitigate adverse effects, thereby improving the quality of patient survival. However, our analysis has some limitations. Recent studies have reported new strategies for combining two drugs to treat tumors; therefore, further mining of data available in databases for assessing the sensitivity exhibited by patients after combining two or more drugs is needed in our subsequent studies (73). In addition, Schürer and his concurrent report that the Clinical Kinase Index can be used as a method to prioritize understudied kinases as drug targets for cancer therapy, providing great value for the development of clinical biomarkers or drug targets (74). Therefore, some clinical kinases should be included in our subsequent studies, which is another limitation of our study. Overall, our study reveals the important role of chemosensitivity in individualized therapy in ccRCC patients, which provides strong support for future clinical practice and development of therapeutic strategies. This finding will hopefully contribute to the development of more precise and effective therapies to benefit patients with ccRCC.

Our investigation, aimed at establishing a prognostic model centered on mitochondrial permeability transition-driven necrosis-associated long non-coding RNAs (LncRNAs), bears significant clinical implications concerning the prognosis and therapeutic strategies for patients afflicted with renal clear cell carcinoma. Additionally, our study yields novel insights into the potential identification of fresh prognostic biomarkers. Nonetheless, it is essential to acknowledge the presence of certain limitations in our research. Firstly, our study relied exclusively on data from the TCGA dataset, and although attempts to validate it via external datasets were made, the availability of valid lncRNA information was hampered by inherent biases and data limitations. Consequently, the validation process demands the incorporation of additional real-world data to reinforce its credibility. Accordingly, forthcoming experiments will be conducted to substantiate the validity of our assertions. Secondly, in pursuit of heightened specificity and accuracy within the prognostic model, we incorporated 15 MPTDNLs as independent prognostic variables for ccRCC. However, we acknowledge that this approach may impose a financial burden on patients. As our research endeavors continue, addressing these limitations will be pivotal to bolster the clinical utility and applicability of our findings in the context of renal clear cell carcinoma management.

## Data availability statement

The original contributions presented in the study are included in the article/**Supplementary Material**, further inquiries can be directed to the corresponding authors.

## Author contributions

JH: Writing – original draft, Conceptualization, Data curation, Formal analysis, Visualization, Writing – review & editing. ML: Writing – original draft, Validation. HC: Data curation, Visualization, Writing – original draft. JZ: Data curation, Visualization, Writing – original draft. XX: Writing – original draft. LJ: Data curation, Visualization, Writing – original draft. SZ: Data curation, Writing – original draft. CJ: Writing – original draft, Writing – review & editing. JieZ: Writing – original draft, Writing – review & editing. QZ: Writing – original draft. GY: Writing – original draft, Writing – review & editing, Conceptualization. HChi: Writing – original draft, Writing – review & editing, Data curation. GT: Conceptualization, Writing – original draft, Writing – review & editing.
