# Supplementary material for: Elucidating the Influence of MPT-driven necrosis-linked LncRNAs on immunotherapy outcomes, sensitivity to chemotherapy, and mechanisms of cell death in clear cell renal carcinoma
Source: Front Oncol. 2023 Dec 15;13:1276715. doi: 10.3389/fonc.2023.1276715 (PMC10757362; doi:10.3389/fonc.2023.1276715)
Supplement: Supplementary file 1 [file Table_1.docx]

**Table S1**. Primers used in qRT-PCR analysis.

| Gene | Sequences (5’-3’) |
| --- | --- |
| H-GAPDH-F | GAACGGGAAGCTCACTGG |
| H-GAPDH-R | GCCTGCTTCACCACCTTCT |
| H-CDK6−AS1-F | TTCTGCTTCCACCTGCTGAT |
| H-CDK6−AS1-R | TGGCAGCTTTCAGGGGTTTT |
